# GRA-PIN: A Graphical and PIN-Based Hybrid Authentication Approach for Smart Devices

**DOI:** 10.3390/s22041349

**Published:** 2022-02-10

**Authors:** Nabeela Kausar, Ikram Ud Din, Mudassar Ali Khan, Ahmad Almogren, Byung-Seo Kim

**Affiliations:** 1 Department of Information Technology, The University of Haripur, Haripur 22620, Pakistan; nabeelawajahat@gmail.com (N.K.); madilator@gmail.com (M.A.K.); 2 Department of Computer Science, College of Computer and Information Sciences, King Saud University, Riyadh 11633, Saudi Arabia; ahalmogren@ksu.edu.sa; 3 Department of Software and Communications Engineering, Hongik University, Sejong 30016, Korea

**Keywords:** authentication, mobile nodes, graphical password, smart devices, software usability

## Abstract

In many smart devices and numerous digital applications, authentication mechanisms are widely used to validate the legitimacy of users’ identification. As a result of the increased use of mobile devices, most people tend to save sensitive and secret information over such devices. Personal Identification Number (PIN)-based and alphanumeric passwords are simple to remember, but at the same time, they are vulnerable to hackers. Being difficult to guess and more user-friendly, graphical passwords have grown in popularity as an alternative to all such textual passwords. This paper describes an innovative, hybrid, and much more robust user authentication approach, named GRA-PIN (GRAphical and PIN-based), which combines the merits of both graphical and pin-based techniques. The feature of simple arithmetic operations (addition and subtraction) is incorporated in the proposed scheme, through which random passwords are generated for each login attempt. In the study, we have conducted a comparative study between the GRA-PIN scheme with existing PIN-based and pattern-based (swipe-based) authentications approaches using the standard Software Usability Scale (SUS). The usability score of GRA-PIN was analyzed to be as high as 94%, indicating that it is more reliable and user friendly. Furthermore, the security of the proposed scheme was challenged through an experiment wherein three different attackers, having a complete understanding of the proposed scheme, attempted to crack the technique via shoulder surfing, guessing, and camera attack, but they were unsuccessful.

## 1. Introduction

The use of smart devices has increased significantly in recent years, and as a result, the amount of sensitive data saved has increased as well. Access to these devices is necessary to avoid the misuse of confidential information. Smartphones, servers, smartwatches, operating systems, and web apps all have mechanisms in order to minimize unauthorized access to critical data and resources. As a result, authentication technologies are essential for enforcing access control regulations and ensuring secure system access.

Authentication is a term used to describe the process of verifying a user’s identity [1]. User authentication is an essential step in using a smart device to access some program or vital data. Many authentication techniques are available that are used to verify the user identity. Some authentication systems, such as simple password authentication, are easy to implement, but in general, they are insecure and rudimentary. The fact that simple textual password authentication is the most extensively used method of authentication attests to the importance of the security flaw [2]. Human considerations such as ease of use and accessibility must also be considered during the developing phases of computer security systems. The core of a security system consists of three interrelated concepts: identification, authentication, and authorization. The following are the different types of authentication mechanisms.

- Authentication using something you are (e.g., Biometrics).
- Authentication using something you know (e.g., Textual Password).
- Authentication using something you process (e.g., Calculation).
- Authentication using something you have (e.g., Token) [3].

Single-factor authentication is the simplest method of authentication. A password (credential) is used to verify an individual’s identity. Most of today’s verification uses this type of authentication. Primarily, personal smart devices and web applications (e.g., social network applications) use one-time user authentication techniques, such as the user name and password technique [4]. Physical tokens (e.g., identity card, credit/debit card, etc.) are also used to enforce access control in physical systems, e.g., opening office or hotel room doors [5]. The biometric identity of the user, e.g., fingerprint or retina, is also used as a common authentication method [6]. These authentication techniques are frequently insufficient for safeguarding personal smart devices, since attackers may quickly retrieve all private data from the device by breaking them [7].

A multi-factor authentication scheme is another way to implement user authentication in which more than one factor (for example, a password and a smart device or biometrics) is involved. An ATM card (credit or debit) with a PIN is commonly used in banking. In some systems, the combination of biometrics with textual passwords is used to authenticate a user. Multi-factor authentication is vulnerable to various attacks, since an attacker can steal these credentials and gain access to a user’s personal information.

**Figure 1:**
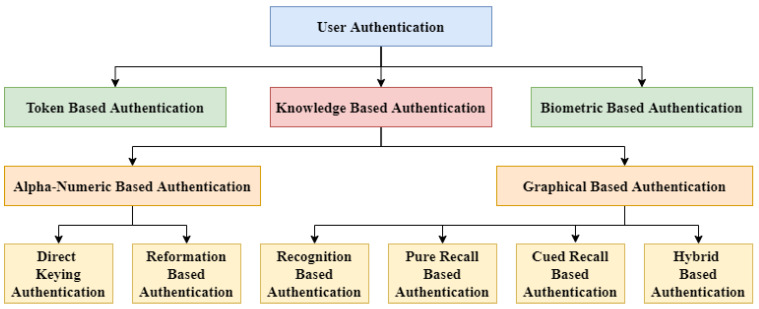
Taxonomy of user authentication techniques.

Authentication techniques are usually classified into three categories: token-based authentication, knowledge-based authentication, and biometric-based authentication. Authentication methods can be divided into one of the three standard approaches, as shown in Figure 1. There is a need to explore graphical passwords, which may include simplicity as well as robustness.

The rest of the article is structured as follows: Section 2 discusses the motivation behind the proposed scheme. Section 3 explains existing literature in respect of authentication schemes. The following section describes the proposed system and its various use case scenarios and GRA-PIN’s working. The next sections explain the research design, testing tool, results, and discussion. The paper ends with a conclusion and possible future directions.

## 2. Related Work

The use of smart devices has exploded in recent years. Before gaining access to the device, the user must be authorized to keep sensitive data protected. Although most people prefer standard alphanumeric passwords, researchers have proposed different authentication approaches to verify a user’s identity over time. We propose a hybrid approach that combines pin and graphical authentication mechanisms.

The authors in [8] suggested a technique in which a mouse signature completes the user’s authentication. The registration and verification stages of this approach are established separately.

In [9], the authors have devised the concept of drawing a secret, allowing users to generate passwords, which will be unique. The user is provided with a 2D grid-based platform for sketching a picture.

In [10], a technique is suggested wherein a user has to choose a triangle that covers a particular area of the image.

The authors in [11] have presented a method in which a user is required to choose an easy shape, i.e., number, alphabet, geometry, or any other random shape of user’s choice.

The proposed authentication scheme in [12] is effective, secure, and useful. In the registration step, first of all, the user has to select a user name and an alphanumerical password. Then, different images can be displayed on the screen, and the user is asked to choose at least three pictures. After selecting the objects, the user uses a stylus or a mouse to sketch them on the screen that can be stored in the database.

The authors in [13] suggested an authentication approach where a picture of a hand is displayed on the screen with *N* rounds of challenges. The number of rounds can be three, four, or five based on the user choice. Furthermore, the login step of the proposed scheme takes more time than other existing graphical techniques. As a result, a more sophisticated authentication scheme should be provided to improve this method.

The authors in [14] suggested a hybrid scheme in which a user has to type a username and an alphanumeric password to be saved in the database.

In [15], the authors have proposed a scheme based on pass faces. It is a two-step process where a picture is merged with text in the first step and text with visuals in the second step.

In [16], a technique is proposed wherein the interface consists of images with text inside. The use of text in pictures rather than graphics enhanced the memory space.

The authors in [17] have proposed 3D passwords, which are flexible, secure, and easier to remember than text-based passwords. Furthermore, these passwords can be effective for different fields, i.e., aeroplane, jet, etc. Moreover, the results are discussed by analyzing the user study conducted. The study showed that the 3D password is secured from a user perspective.

In [18], a scheme is proposed in which the user identity is identified at the PassBYOP terminal. In addition, a pre-selected password image is placed at the camera of PassBYOP and the captured image is displayed on the adjacent screen. At the third level, the password location in an image is matched.

PassBYOP provides additional security at the mobile phone level to secure the system from theft [18]. Due to easy usability, users can take measures against password removal in the stolen device. PassBYOP, in combination with other entropy calculation techniques, can be useful for providing security against brute force attacks. In the observation phase, shoulder surfing, camera, and malware attacks are considered where the password is difficult to guess. The proposed strategy is advantageous and efficient for providing security against shoulder-surfing attacks.

Furthermore, the evaluation of the proposed method is determined by reliability, usability, and security perspectives by designing a prototype. For reliability analysis, different users are participated at a terminal level for collecting images, and SIFT is used for feature matching [19]. Two factors, i.e., entry time and error rates, are used for usability analysis. Different participants are involved in conducting the study. The results revealed that users are satisfied with this authentication approach and find it challenging to the exact location of the image. Theft, guessing, and observation attacks are considered from a security perspective.

In [20], an approach is proposed to prevent shoulder surfing attacks without compromising system usability.

The authors in [21] have suggested an authentication mechanism for web applications. The proposed methodology is based on sending an alphanumeric password to the user’s registered email, and then, three images on the user interface are used for setting a password.

In [22], a scheme is proposed wherein multiple combinations of pictures with various shapes and colors are used. Some other recent works have also been performed in the context of the graphical user authentication side. The authors in [23] have proposed a gamified version of the graphical password, GamePass, that is tested to be less predictable and effective for nudging users. They have left open directions to evaluate the GamePass techniques with other graphical user authentication techniques and incorporate aspects such as memorability and security to validate their findings further.

Another recent work performed in [24] proposes a recall-based graphical password that uses the help of permutation, a mathematical technique to determine the number of possible arrangements, to form a graphical triangle. The second phase of the authentication technique prompts the user to enter a one-time password sent to the user. The contributions and limitations of the existing techniques are provided in Table 1.

## 3. Motivation

Many users have tended to save their confidential data on smartphones in recent years, and such devices are now cheap and readily available. Mostly, users use a textual password for authentication as it is easy to remember, fast, and cost-effective. This traditional authentication technique is vulnerable to shoulder surfing attacks, dictionary attacks, malware attacks, and camera attacks [26]. Strong textual passwords provide security but are challenging to remember. To date, none of the existing research claims with conviction about a foolproof secure authentication protocol for smart devices. Greg Blender defined the notion for the graphical password in 1996, and numerous systems for the authentication of the graphical password were developed afterwards [16]. Graphical passwords are attractive because images are easy to remember. This study proposes a hybrid authentication technique that combines textual and graphical password authentication methods to increase usability and security. The proposed approach is very secure to shoulder surfing attacks, guessing attacks, and camera attacks [27], as the new password will be generated every time a user will log in.

The main goal of the proposed authentication scheme is to figure out how to develop a new hybrid password strategy that combines usability and security. It can be further divided into the following four objectives.

- Create a novel hybrid password technique providing usability and security in parallel.
- Integrate PIN method with graphics and arithmetic operations generating every time a new password.
- Evaluating the security of our proposed technique using different approaches.
- Evaluating and comparing our proposed authentication technique with existing ones using different standards and statistical model.

While protecting sensitive data and devices, text passwords were exhausted. Alphanumeric passwords were a hybrid strategy that protected against different threats for a while but also had a number of drawbacks. This research work introduces a graphical and PIN-based password scheme that improves user security without losing usability. The proposed GRA-PIN authentication scheme is evaluated through the software usability scale (SUS) questionnaire. The SUS questionnaire results show that the overall results for the proposed scheme are good, as students opted for a 94 SUS score. This novel authentication scheme will be a milestone in the field of knowledge-based authentication.

## 4. Material and Methods

User authentication techniques are enforced to secure devices and essential data from illegal access. Several approaches have been offered, including text-based passwords, biometric-based approaches, and arranging images/icons or allowing users to register pictures of their own choice. Text-based passwords are easy to remember but less secure, while graphical passwords are difficult to crack but require more space. The shoulder surfing attack is also a significant issue in authentication techniques. Only a few knowledge-based approaches are available to combat shoulder surfing. We have combined the pros of both text-based and graphical authentication techniques and proposed a hybrid approach. This section will further elaborate the working of the proposed architecture and highlight steps involved in various important phases such as registration and authentication.

### 4.1. Implementation

To implement the proposed technique, we have used the following tools and technologies: Android Flutter 1.17.0 by Google and Android Studio Emulator.

The proposed system has two phases, i.e., registration and authentication, which are discussed below.

### 4.2. Registration Phase

**Figure 2:**
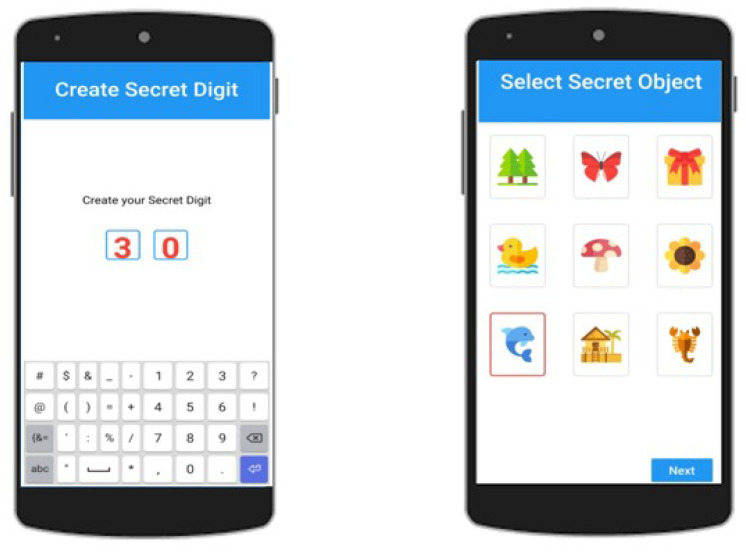
Select 2-digit secret number and one secret image.

**Step 1:** A user has to select a two-digit Secret Number ranging from 10 to 90 (as shown in Figure 2).

**Step 2:** One Secret Image is selected out of 9 different images.

**Figure 3:**
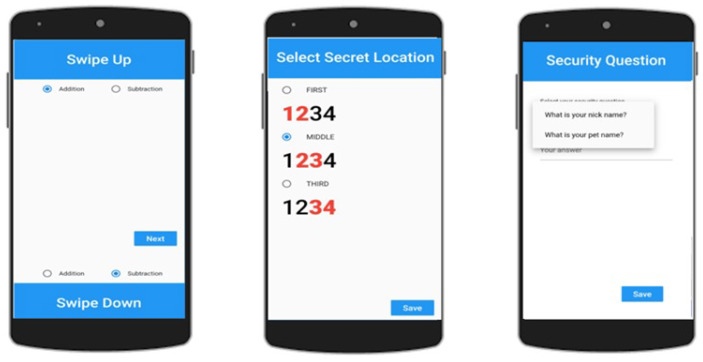
Select arithmetic operation, secret position, and security question.

**Step 3:** The Swipe-Up or Down positions are selected for operator selection. There are only two arithmetic operations, i.e., addition and subtraction (see Figure 3).

**Step 4:** A Secret Position in a 4-digit PIN from the first, middle, and last positions are selected, as **12**34, 1**23**4, and 12**34**, respectively.

**Step 5:** A security question is answered in this step. However, if the password is forgotten, then the user has to answer the security question correctly in order to reset his password again.

### 4.3. Authentication Phase

**Figure 4:**
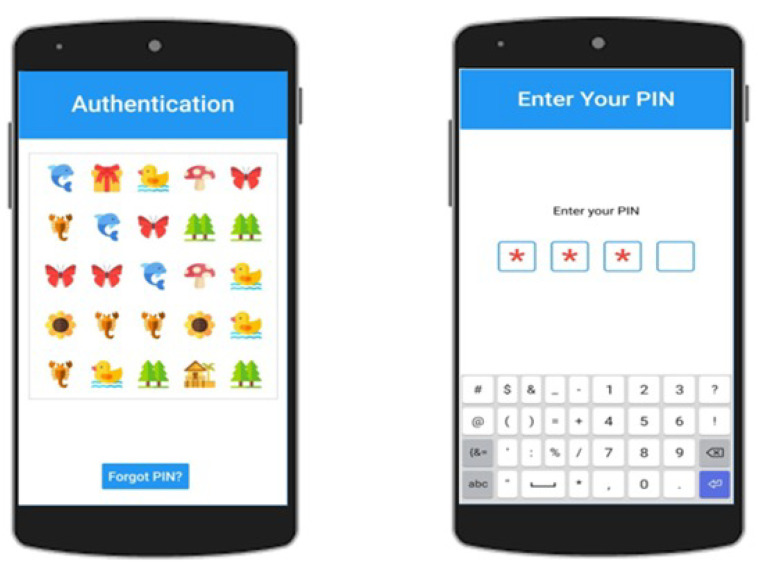
Count pre-selected images and enter the PIN.

**Step 1:** During the authentication phase, a 5 × 5 grid of images will appear on the screen, as shown in Figure 4. The user has to count the secret image that was selected at the Registration Phase. Every image will appear no more than nine times in the grid.

**Step 2:** After counting the selected image, the user has to swipe-up or down the grid to perform either addition or subtraction as registered.

**Step 3:** The user adds/subtracts the secret number to the number of times the secret image was displayed in the grid.

**Step 4:** Entering PIN code: The resultant two-digit number will be placed in the Secret Position; the remaining two input numbers will be RANDOM and discarded during the matching phase, as shown in Figure 4.

**Step 5:** If the user has entered the right password, he will be authenticated and access the device.

**Step 6:** If the user enters a wrong PIN, he must re-enter the PIN. Otherwise, the access will not be granted until the right PIN has been entered.

**Step 7:** If a user forgets his password, he has to answer the security question to reset the password.

Algorithms 1 and 2 show the registration and authentications phases of the proposed system, respectively. We have also mentioned the technologies and tools used to implement the proposed approach.
**Algorithm 1:** Registration Phase
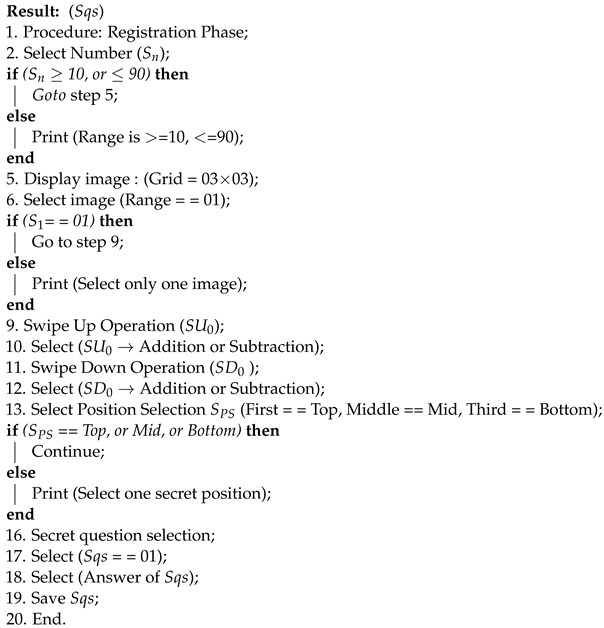

**Algorithm 2:** Authentication Phase
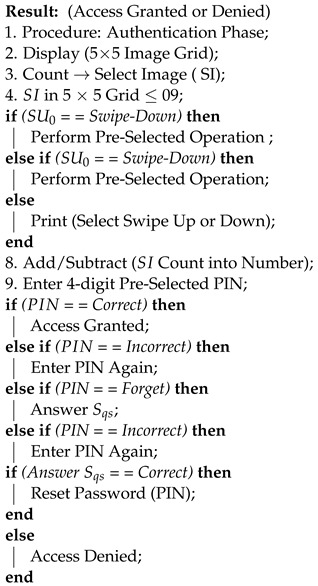


## 5. Proposed GRA-PIN

Considering all the issues and limitations in graphical and pin-based systems, we suggested a hybrid authentication technique combining text and graphical-based authentication techniques. In the GRA-PIN method, users have to select four different options to create a password. Categories of GRA-PIN are a selection of two-digit numbers, selection of one secret image, selection of swipe-up/down position for arithmetic operation and at last selection of password position in the final four-digit PIN. The user also has to answer a secret answer in case of forgetting the password. Every time the user logs in, a new password will be generated, which makes it secure against shoulder surfing, guessing, and camera attacks. Our proposed authentication technique is more reliable, secure, and user-friendly while maintaining usability and security.

### 5.1. The GRA-PIN Framework

This section covers the use case diagram, data flow diagram, and algorithm for the proposed hybrid authentication technique. The use case diagram shows what a user can do with GRA-PIN, whereas the data flow diagram shows how data flows through the application. The algorithm depicts the GRA-PIN scheme’s step-by-step approach.

### 5.2. Conceptual Design of GRA-PIN

The illustrates the requirements of proposed system and desired features. We have three actors in this prototype: an administrator, a new user, and an existing user.

#### 5.2.1. Use Case Diagram for Administrator

**Figure 5:**
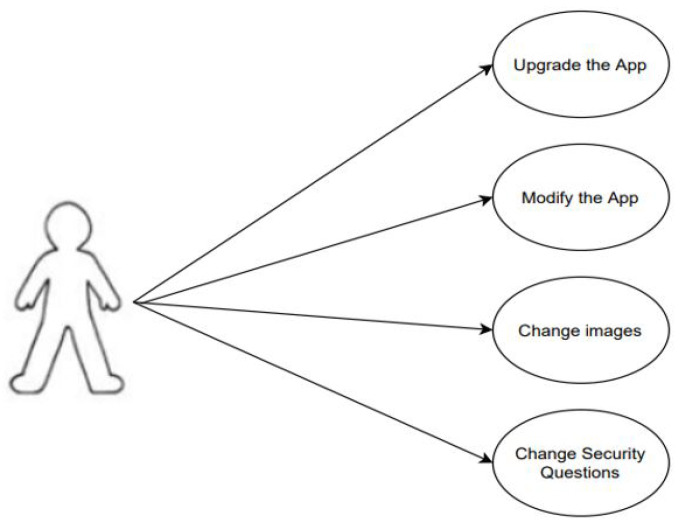
Use case diagram for admin.

The administrator controls and manages the application. The administrator can perform the following activities shown in Figure 5. These activities are upgrading the application, modifying the images, and selecting the security questions.

#### 5.2.2. Use Case Diagram for a New User

**Figure 6:**
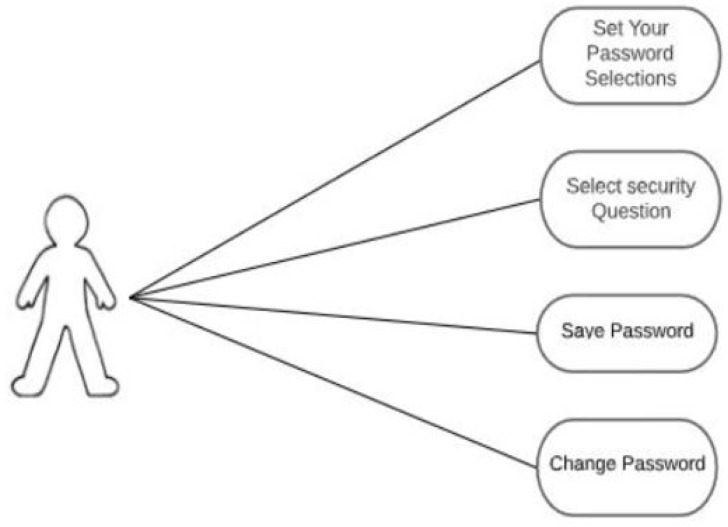
Use case diagram for a new user.

The new user has several activities that can be done on the application; as depicted in the use case diagram in Figure 6, the new user has some activities that can be done on the application. Choosing different categories to form a password, selecting security questions, saving passwords, and changing passwords are examples of these tasks.

#### 5.2.3. Use Case Diagram for an Existing User

**Figure 7:**
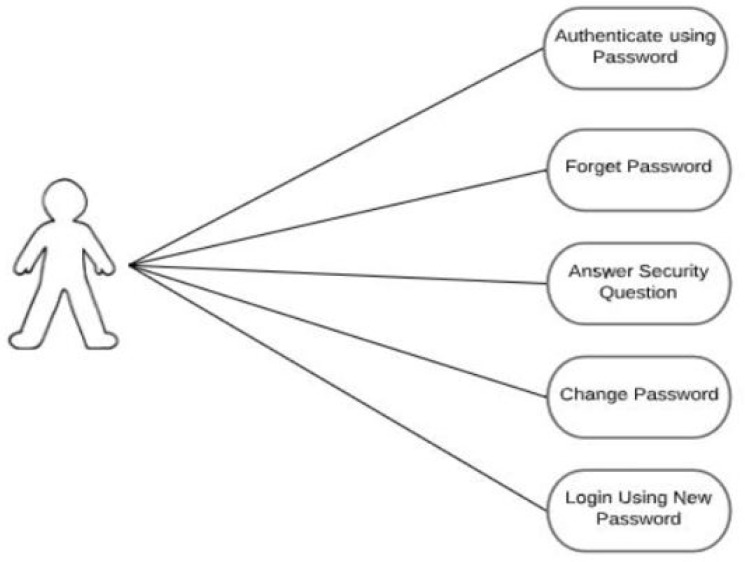
Use case diagram for an existing user.

Figure 7 shows an existing user who has to accomplish something with the prototype. Authenticating using an existing password, changing a password in the case of forgetfulness, and changing a category are examples of these tasks.

The sequence of steps is given below:

(i) A new user creates an account.
(ii) The user selects a 2-digit secret number.
(iii) The user selects one image from 9 different images.
(iv) The user selects arithmetic operators (addition and subtraction) for swipe up/down.
(v) The user selects the position of the password in a 4-digit PIN.
(vi) The password will be saved on the device database.
(vii) The user views the login indicator.
(viii) The user gets access to the smart device using the login indicator.
(ix) The access privilege will be granted to the user.
(x) After using the device, the user can log out.
(xi) If the user forgets his/her password, they click on the Forgot PIN button.
(xii) The user has to answer the security question correctly.
(xiii) The user will reset his password.
(xiv) The user can log into the device with the new password.

### 5.3. System Design

**Figure 8:**
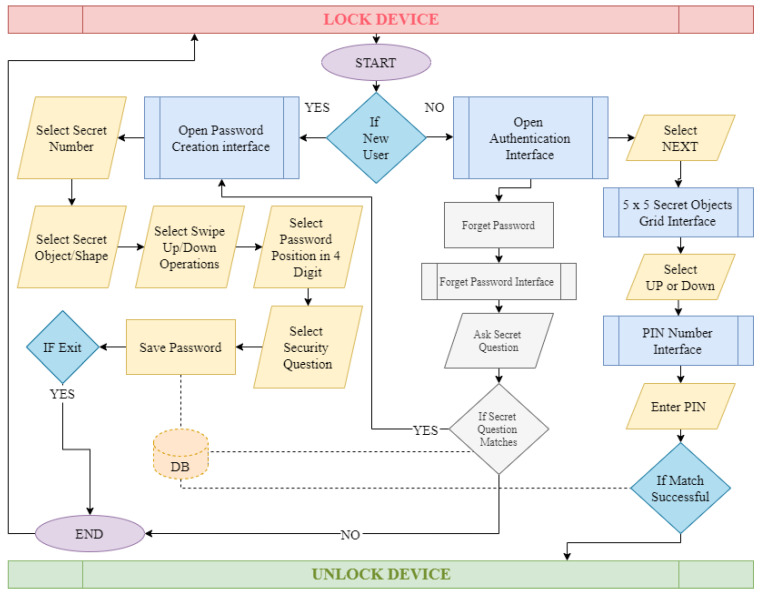
Data flow diagram of GRA-PIN.

The data flow diagram exhibits how the program works for both new and existing users. Figure 8 depicts the design framework of the proposed system.

## 6. Results and Discussion

The formation of a research design is the most important aspect of the entire research study, since it involves many different types of decisions that might lead to a productive and acceptable study. As this is an experimental study, group selection and experimentation were essential factors to consider. Before explaining our proposed design, we should briefly introduce the aspects covered in the designing procedure.

It is a challenging process to evaluate any system for the sake of validating its core criteria and objectives as well as choosing a suitable assessment model [28]. In 2008, Fraenkel and Wallen released a study that gives an in-depth understanding of the research, experimentation, statistical measurement, and evaluation.

### 6.1. Research Design

Group selection and experimentation were the key factors to consider because this was an experimental study. For this study, we adopted the randomized post-test-only research design with two groups, and participants are chosen randomly.

Group X will be referred to as the treatment group, which will perform the tasks on the proposed scheme. The second group C will assign the tasks on existing screen lock applications, and both groups would be tested for dependent variables O in the same way. Internal validity may be compromised by such a research design; however, statistical regression, subject characteristics, and maturation risks are mitigated by allocating participants in randomized order.

### 6.2. Sampling

A sample approach is selecting a small group of people as representatives of the entire population to derive conclusions about the whole. The sampling method describes how observations are obtained from a population to be included in a sample study [29]. On a random sample of participants, a user-centric evaluation of the study was conducted. The proposed study was separated into two groups based on randomized post-test-only research methodology: control and treatment groups. The questionnaire will ask questions in order to collect relevant data and feedback from users. The experiment is carried out with a total of 30 users.

- **Control Group:** This group consisting of 20 participants were selected randomly from Computer Science Department at Govt. Postgraduate College for Women, Haripur. They were required to complete some tasks as well as fill out a questionnaire. The control group was bound to do the tasks on their own Android smartphone, which must have a screen lock application installed. The activities were completed by 10 participants of group G1 using the Android pattern lock application and 10 participants of group G2 using the Android PIN lock application.
- **Treatment Group:** The treatment group G3 consisted of ten randomly selected participants to perform the given tasks using the suggested GRA-PIN authentication prototype. The group was initially given a PowerPoint presentation regarding the system, which included a detailed explanation of the prototype’s interfaces. After that, participants were given tasks to complete, which were followed by a questionnaire for the prototype. The treatment group had to get the prototype, which was installed on certain smartphones, and the study was conducted at the same department.

### 6.3. Analysis Process

In 1996, John Brooke proposed the System Usability Scale (SUS) to evaluate the usability of different artifacts, which is reliable and cost-effective [30]. The SUS is selected to evaluate the GRA-PIN scheme regarding its effectiveness, user satisfaction, ease of use, usefulness, and interactivity of the authentication techniques. So, instead of designing the questions individually for all these parameters, SUS covers all of them in just 10 statements, which are listed as follows:

Statement 1: “*I think I would like to use this system frequently.*”

Statement 2: “*I found the system unnecessarily complex.*”

Statement 3: “*I thought the system was easy to use.*”

Statement 4: “*I found that I would need the support of a technical person to be able to use this system.*”

Statement 5: “*I found the various functions in this system were well integrated.*”

Statement 6: “*I thought there was too much inconsistency in this system.*”

Statement 7: “*I would imagine that most people would learn to use the system very quickly.*”

Statement 8: “*I found the system very cumbersome to use.*”

Statement 9: “*I felt very confident using the system.*”

Statement 10: “*I needed to learn a lot of things before I could get going with this system.*”

This can save a user’s time in contrast with another questionnaire, so the possibility of getting an accurate response from the user is much more. The SUS is made up of ten questions with answers ranging from Strongly Disagree (SD) (1) to Strongly Agree (SA) (5). For questions having odd question number, the score is calculated as the scale value minus one, i.e., Strongly Disagree (SD) is considered as 0, whereas Strongly Agree (SA) is considered as 4. Questions have an even number, as questions are calculated by subtracting each value from 5. Thus, Strongly Disagree (SD) is considered as 4, whereas Strongly Agree (SA) is considered as 0. Each row represents the opinion of all the participants about one particular statement. For example, in Table 2, all the 10 participants rated 5 in response to odd questions such as 1 and 9; therefore, the average score is evaluated as [(5−1)×10)/10]=4. Similarly, for statement 2, six participants voted 1, and two users voted 2. Therefore, the average score will be calculated as [((5−1)×6)+((5−2)×4)]/10=3.6. After calculating the score of each question, the addition of all scores will lead us to the overall average score. The total score of SUS will be calculated by multiplying the sum of the scores by 2.5. The range of SUS scores is 0 to 100.

### 6.4. Usability

Usability is the relationship between the users and system that determines the quality of interactivity, which enables the ease of use to achieve defined goals with efficacy and satisfaction in the context in which a system or method is employed [31]. Usability can be decomposed into measurements and sub-measures such as effectiveness, learnability, task completion time, and satisfaction, among others [32].

The students chose the best options in favor of the GRA-PIN authentication application, which had an SUS usability score of 94, according to the results of the SUS questionnaire displayed in Table 2. Students’ responses to questions 1 and 9 indicate that they are all satisfied with the proposed authentication scheme. The answers to questions 2, 3, 7, and 8 indicate that the suggested design is user-friendly. The proposed scheme is highly integrated, according to the results of question 5 and 6, and the application’s learnability is excellent according to the results of questions 4 and 10.

As indicated in Table 3, the overall results for the PIN lock application are average, with students opting for an SUS score of 69.5. As a result of their dissatisfaction, the students demonstrated less trust in their given applications. Table 4 shows the SUS questionnaire findings of students who used the pattern lock application, which show an SUS score of 60.

### 6.5. Statistical T-Test Evaluation

We have used a statistical *t*-test [19] to evaluate whether the difference between both the groups are statistically significant or not and how much difference exists between both approaches. For that purpose, we have formulated two null hypotheses and then conducted two *t*-tests for both. As described in Table 5, our first comparison was between G1 and G3, and it is evident from the results that we can reject the null hypothesis and can say that GRA-PIN based authentication shows a significantly better average score for usability than the pattern-based authentication, t (9.911) = 4.834, *p* = 0.001.

Similarly, Table 6 shows the results for the second comparison that was between G2 and G3. GRA-PIN-based authentication results are significantly better in terms of the average score of usability than PIN-based authentication, t (18) = 8.092, *p* < 0.01.

### 6.6. Security

GRA-PIN appears to have promise in terms of security against shoulder surfing, camera attack, and guessing attacks.

**(a)**  **Participants:** This study had three participants (attackers), which is a standard number for this type of experiment.
**(b)**  **Procedure:** A brief PowerPoint presentation on the GRA-PIN authentication technique was delivered to all three participants. The attackers must use shoulder-surfing, camera, and guessing attacks to crack the password. At least a 20-min break will separate each attack attempt. The attackers were given 20 min and five chances to type the accurate password. If the attacker succeeds in cracking the password within the specified time, he will go on to the next attack type. If all five attempts are unsuccessful, they will continue to the next attack type.

For a shoulder-surfing attack, the victim will log in to his device three times in front of attackers, and they will observe the activity within a distance of 2 m. The attackers are allowed to take notes in order to crack the password.

Attackers were allowed to record a video from any recording device of their choice within 1.5 m of the victim in a camera attack. The footage shows the entire login process, including the four-digit PIN entering.

In the guessing attack, a brief description of the GRA-PIN approach was given to all three participants (attackers) and they were allowed to guess the password. During the attacks, attackers were free to utilize whatever tools or resources they wished. The experiment took about 2 h for each participant to complete.

Attacks on GRA-PIN took extensive time and effort, but no attacker managed to crack GRA-PIN passwords. In the proposed scheme, a new password will be generated every time the user logs in to the device, which demonstrates the increased security of the GRA-PIN approach against attacks. This shows that if the GRA-PIN authentication scheme is used in a real-world scenario, it would be highly resistant to these attacks.

### 6.7. Discussion

To summarize the overall comparison of the proposed GRA-PIN authentication system with two alternate approaches, i.e., PIN lock and pattern lock, GRA-PIN performed well. The SUS score of GRA-PIN was considerably high, 94. At the same time, PIN lock had an SUS score of 69.5 and pattern lock of 60.

As far as the findings of the *T*-test are concerned, GRA-PIN had a comparatively better average score of usability compared to pattern-based authentication, t (9.911) = 4.834, *p* = 0.001.

Furthermore, GRA-PIN-based authentication results were also significantly better for usability compared to PIN-based authentication, t (18) = 8.092, p< 0.01.

Moreover, all the attacks attempted on GRA-PIN took time and effort with no success ratio at all.

### 6.8. Future Work

As GRA-PIN is a novel technique that incorporates various levels of authentication, which are simple yet hard to break, many future directions contained can further enhance the security: for example, the use of hardware-based technologies such as an accelerometer, touch or fingerprint sensor, GPS, microphone, etc., in any level of the authentication. In most of the cases, a trade-off exists between usability and security, which is supposed to be maintained at its due level.

## 7. Conclusions

The GRA-PIN authentication scheme is a hybrid technique that combines textual and graphical authentication techniques. A new feature, i.e., simple arithmetic operations (addition and subtraction), is hidden in the proposed scheme. A new password will be generated every time the user logs in to the device, making it more secure against attacks. A randomized post-test-only study is conducted to examine GRA-PIN’s usability. Thirty participants were distributed into two groups, i.e., treatment and control groups. The proposed scheme is evaluated for its usability using a standard System Usability Scale (SUS), which is actually a ten-item Likert Scale. The participants were assigned the tasks and questionnaire to conduct the study. The results of the questionnaire were compared through T-test, where the analysis shows that the GRA-PIN authentication scheme is effective, easy to use, and user friendly. The security of the GRA-PIN technique is evaluated through an experimental approach in which three participants (attackers) used shoulder surfing, camera attack, and guessing attack to crack the password but could not type the correct password.

People usually choose long passwords to secure them against shoulder-surfing attacks, but long passwords are difficult to remember. The GRA-PIN approach generates a two-digit password, which is very difficult to hack. Overall, our proposed scheme is highly secure, reliable, and user friendly.

## Figures and Tables

**Table 1:** Comparison of existing techniques.

Ref.	Technique	Contribution	Limitation
[8]	Use of mouse/keyboard to draw a signature	Easy to remember	Guessing attack, shoulder surfing attack, and dictionary attack
[9]	Draw a secret (DAS)	Easy to draw	Drawing sequence is hard to remember
[10]	Click within a region delimited by pre-registered images	Secure against shoulder surfing attack	When a huge number of objects are involved, it can be difficult to remember
[11]	Draw a selected shape in grid	Easy to remember	Shoulder surfing attack
[12]	Draw a sketch of pre-selected images	Secure against brute force, spyware, and dictionary attack	Time consuming
[13]	A picture of a hand will be displayed with N rounds	Secure against shoulder surfing attack	Login phase takes more time
[14]	Select a maximum of four objects (symbols/shapes)	Easy to remember	Shoulder surfing attack
[15]	Recognize and pick the pre-registered pictures	Although faces are simpler to recall, the options are still predictable	Brute force, dictionary attack, and shoulder surfing attack
[16]	Use of text in six alphabets in proposed layout	Useful in terms of space utilization	Shoulder surfing attack
[17]	3D environment-based authentication	Flexible, easy to remember	Weak against guessing attack
[18]	Use of PassBYOP terminal	Secure against shoulder surfing attack	Registration phase takes time
[20]	Pass-Matrix authentication	Secure against shoulder surfing attack	Login phase takes time
[21]	Use of graphical password for web-based applications	Easy to remember	Shoulder surfing attack
[22]	Graphical-based scheme	Hard to crack	Difficult to remember
[25]	Double grid-based graphical pattern	More usable and secured	Less memorable and satisfying

**Table 2:** SUS Results of G3 (GRA-PIN authentication application users). Average SUS Score = 37.6, Total SUS Score = 37.6 × 2.5 = 94.

S.No	Concerned Statement	SD (1)	2	3	4	SA (5)	Avg Score
1	Statement 1	0	0	0	0	10	4
2	Statement 2	6	4	0	0	0	3.6
3	Statement 3	0	0	0	5	5	3.5
4	Statement 4	8	0	2	0	0	3.6
5	Statement 5	0	0	0	1	9	3.9
6	Statement 6	9	1	0	0	0	3.9
7	Statement 7	0	0	0	5	5	3.5
8	Statement 8	8	2	0	0	0	3.8
9	Statement 9	0	0	0	0	10	4
10	Statement 10	8	2	0	0	0	3.8

**Table 3:** SUS results of G1 (Android pattern lock application users). Average SUS Score = 24, Total SUS Score = 24 × 2.5 = 60.

S. No	Concerned Statement	SD (1)	2	3	4	SA (5)	Avg Score
1	Statement 1	1	5	2	2	0	1.5
2	Statement 2	0	1	5	4	0	1.7
3	Statement 3	0	1	1	6	2	2.9
4	Statement 4	7	3	0	0	0	3.7
5	Statement 5	1	3	5	1	0	1.6
6	Statement 6	0	3	6	1	0	2.2
7	Statement 7	0	0	3	5	2	2.9
8	Statement 8	2	6	2	0	0	3
9	Statement 9	5	1	1	3	0	1.2
10	Statement 10	4	5	1	0	0	3.3

**Table 4:** SUS Results of G2 (Android PIN lock application Users). Average SUS Score = 27.8, Total SUS Score = 27.8 × 2.5 = 69.5.

S.No	Concerned Statement	SD(1)	2	3	4	SA(5)	Avg Score
1	Statement 1	0	2	3	4	1	2.4
2	Statement 2	3	3	2	2	0	2.7
3	Statement 3	0	0	1	6	3	3.2
4	Statement 4	6	2	1	1	0	3.3
5	Statement 5	0	0	5	3	2	2.7
6	Statement 6	2	4	3	1	0	2.7
7	Statement 7	1	0	2	4	3	2.8
8	Statement 8	2	4	3	0	1	2.6
9	Statement 9	0	5	0	2	3	2.3
10	Statement 10	4	3	3	0	0	3.1

**Table 5:** *T*-test evaluation for G1 and G3.

Variable	G3 (GRA-PIN) Mean	G1 (Pattern) Mean	*t*-Value	Effect Size	*p*-Value	95% CI For Mean Difference
SUS Average Score	3.760	2.400	4.834	2.161	0.001	0.7689, 1.9511

**Table 6:** *T*-test evaluation for G2 and G3.

Variable	G3 (GRA-PIN) Mean	G2 (PIN) Mean	*t*-Value	Effect Size	*p*-Value	95% CI For Mean Difference
SUS Average Score	3.760	2.780	8.092	3.618	<0.01	0.7256, 1.2344

## Data Availability

Not applicable.
